# A Bit of Humor

**Published:** 1876-10

**Authors:** 


### *A BIT OF HUMOR.*

Our neighbors of the Health Food Company send out immense quantities of entertaining printed matter concerning their choice foods. Applications for circulars reach them by the hundred weekly, and some of the communications comprise very interesting accounts of the miseries experienced by the writers. Here is a copy of one of another sort :

"Please send me your pamphlet on 'Health Food.' Augustus Skean, Crooked Hill, Montgomery County, Pa."

Then followed this verse :

"Food for health and health for food,
We must have both or get no good ;
We ask for health and 'tis not doubted,
That by your foods disease is routed ;
But never mind, send us the book ;
We'll eat and live ! thus says our cook."

To this our ready neighbors sent the following response :

"Your poetical demand for printed matter relating to Health Foods is at hand, and we mail you a good supply. We shall have great hopes of you as soon as you begin to live generously.

"Your Crooked Hill shall straightened be,
Your labors not in vain,
If upon Healthy Food you live,
Oh, friend! Augustus Skean.

"Eat Gluten, but no glutton be;
Take food that gives you brain;
Thus shall you long, sweet verses write—
Dear friend! Augustus Skean."

HEALTH FOOD COMPANY.

